# *N*-(1*H*-1,2,4-Triazol-5-yl)pyridine-2-carboxamide

**DOI:** 10.1107/S1600536809041269

**Published:** 2009-10-17

**Authors:** Jing Miao, Maomao Jia, Xianlin Liu, Wei Xiong, Zilu Chen

**Affiliations:** a College of Chemistry and Chemical Engineering, Guangxi Normal University, Yucai Road 15, Guilin 541004, People’s Republic of China

## Abstract

In the structure of the title compound, C_8_H_7_N_5_O, the pyridine ring and the imidazole ring are nearly coplanar, making a dihedral angle of 2.97 (15)°. An intra­molecular N—H⋯O hydrogen bond occurs. In the crystal mol­ecules are connected by inter­molecular hydrogen bonds and π–π stacking inter­actions between neighboring imidazole rings [centroid–centroid distance = 3.5842 (5) Å and off-set angle = 21.77°], leading to the formation of a two-dimensional supra­molecular sheet.

## Related literature

For an alternative preparative method for the title compound, see: Browne & Polya (1968 ▶). For the potential bioinorganic applications of 1,2,4-triazole derivatives, see: Bohm & Karow (1981 ▶); Bahel *et al.* (1984 ▶).
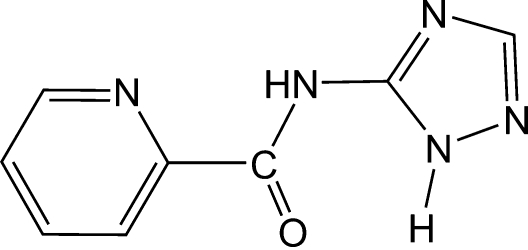


## Experimental

### 

#### Crystal data

C_8_H_7_N_5_O
*M* *_r_* = 189.19
Monoclinic, 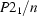

*a* = 8.6906 (17) Å
*b* = 5.2854 (10) Å
*c* = 17.880 (4) Å
β = 90.700 (3)°
*V* = 821.2 (3) Å^3^
*Z* = 4
Mo *K*α radiation
μ = 0.11 mm^−1^
*T* = 273 K
0.26 × 0.24 × 0.18 mm

#### Data collection

Bruker APEXII CCD area-detector diffractometer
Absorption correction: multi-scan (*SADABS*; Bruker, 1998 ▶) *T*_min_ = 0.972, *T*_max_ = 0.980
3938 measured reflections
1443 independent reflections
961 reflections with *I* > 2σ(*I*)
*R*_int_ = 0.095

#### Refinement

*R*[*F* ^2^ > 2σ(*F* ^2^)] = 0.046
*wR*(*F* ^2^) = 0.106
*S* = 1.00
1443 reflections
128 parameters
H-atom parameters constrained
Δρ_max_ = 0.17 e Å^−3^
Δρ_min_ = −0.16 e Å^−3^

### 

Data collection: *APEX2* (Bruker, 2004 ▶); cell refinement: *SAINT* (Bruker, 2004 ▶); data reduction: *SAINT*; program(s) used to solve structure: *SHELXS97* (Sheldrick, 2008 ▶); program(s) used to refine structure: *SHELXL97* (Sheldrick, 2008 ▶); molecular graphics: *SHELXTL* (Sheldrick, 2008 ▶); software used to prepare material for publication: *SHELXTL*.

## Supplementary Material

Crystal structure: contains datablocks global, I. DOI: 10.1107/S1600536809041269/ez2185sup1.cif

Structure factors: contains datablocks I. DOI: 10.1107/S1600536809041269/ez2185Isup2.hkl

Additional supplementary materials:  crystallographic information; 3D view; checkCIF report

## Figures and Tables

**Table 1:** Hydrogen-bond geometry (Å, °)

*D*—H⋯*A*	*D*—H	H⋯*A*	*D*⋯*A*	*D*—H⋯*A*
N2—H21⋯N3^i^	0.88	2.09	2.946 (2)	164
N4—H41⋯O1^ii^	0.86	2.06	2.873 (2)	158
N4—H41⋯O1	0.86	2.17	2.629 (2)	113

Symmetry codes: (i) 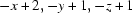; (ii) 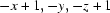.

**Fig. 1.**
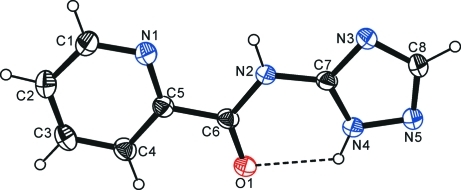
The molecular structure of the title compound with the atom-numbering scheme and 30% displacement ellipsoids.

**Fig. 2.**
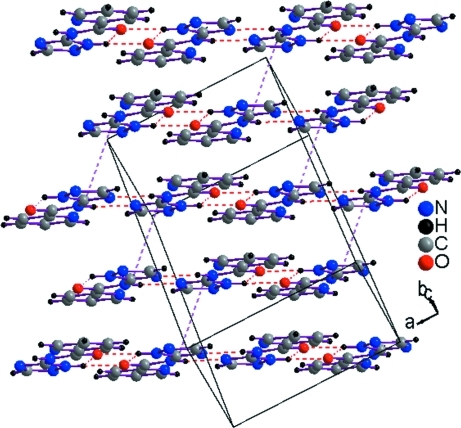
A view of the two-dimensional supramolecular sheet assembled by hydrogen bonds and π–π stacking interactions (indicated by dashed lines).

## supplementary crystallographic information

### Comment

1,2,4-Triazoles derivatives represent an interesting class of heterocycles. They present various potential applications in bioinorganic chemistry (Bohm & Karow, 1981; Bahel, *et al.*, 1984). The preparation of the title compound has been reported previously (Browne & Polya, 1968), but its crystal structure has not yet been reported. Thus we report here the structure (Fig. 1) of the title compound obtained using an alternative method.

The pyridine ring and the imidazole ring are nearly co-planar with a dihedral angle of 2.97 (15)°. An intramolecular N—H···O hydrogen bond is present in the molecule. Adjacent molecules are connected alternatively by intermolecular N—H···N and N—H···O hydrogen bonds into one dimensional supramolecular chains (Fig. 2). The neighboring imidazole rings from adjacent one dimensional chains are parallel to each other with a perpendicular distance of 3.3285 (1) Å, a centroid-to-centroid distance of 3.5842 (5) Å and an off-set angle of 21.774° (calculated as the angle formed by the line through the two centroids of the two imidazole rings and the normal of the imidazole plane). This indicates the presence of a π–π stacking interaction between the neighboring imidazole rings from adjacent one dimensional chains, which leads to the construction of a two dimensional supramolecular sheet (Fig. 2).

### Experimental

A mixture of 1,2-di-2-pyridyl-ethane-dione (0.2122 g, 1 mmol), 5-amino-1,2,4-triazole (0.1682 g, 2 mmol) and methanol (20 ml) was refluxed at 343 K for three hours. It was then filtered and the filtrate was left at ambient temperature to evaporate for three days, yielding crystals of the product. The overall yield is 70%. Elemental analysis for C_8_H_7_N_5_, calculated: C 55.48, H 4.07, N 40.44%; found: C 55.12, H 4.35, N 40.82%.

### Refinement

H atoms on the N atoms were located in an electron density map and and allowed to ride on the N atoms with *U*_iso_(H) = 1.5*U*_eq_(N). H atoms on the carbon atoms were placed at calculated positions (C–H = 0.93 Å) and were included in the refinement in the riding model approximation, with *U*_iso_(H) = 1.2*U*_eq_(C).

### Crystal data

**Table:**

C_8_H_7_N_5_O	*F*(000) = 392
*M**_r_* = 189.19	*D*_x_ = 1.530 Mg m^−^^3^
Monoclinic, *P*2_1_/*n*	Mo *K*α radiation, λ = 0.71073 Å
Hall symbol: -P 2yn	Cell parameters from 1623 reflections
*a* = 8.6906 (17) Å	θ = 3.0–28.0°
*b* = 5.2854 (10) Å	µ = 0.11 mm^−^^1^
*c* = 17.880 (4) Å	*T* = 273 K
β = 90.700 (3)°	Block, colorless
*V* = 821.2 (3) Å^3^	0.26 × 0.24 × 0.18 mm
*Z* = 4	

### Data collection

**Table:**

Bruker APEXII CCD area-detector diffractometer	1443 independent reflections
Radiation source: fine-focus sealed tube	961 reflections with *I* > 2σ(*I*)
graphite	*R*_int_ = 0.095
φ and ω scans	θ_max_ = 25.0°, θ_min_ = 2.3°
Absorption correction: multi-scan (*SADABS*; Bruker, 1998)	*h* = −8→10
*T*_min_ = 0.972, *T*_max_ = 0.980	*k* = −6→5
3938 measured reflections	*l* = −21→21

### Refinement

**Table:**

Refinement on *F*^2^	Secondary atom site location: difference Fourier map
Least-squares matrix: full	Hydrogen site location: inferred from neighbouring sites
*R*[*F*^2^ > 2σ(*F*^2^)] = 0.046	H-atom parameters constrained
*wR*(*F*^2^) = 0.106	*w* = 1/[σ^2^(*F*_o_^2^) + (0.0259*P*)^2^] where *P* = (*F*_o_^2^ + 2*F*_c_^2^)/3
*S* = 1.00	(Δ/σ)_max_ < 0.001
1443 reflections	Δρ_max_ = 0.17 e Å^−^^3^
128 parameters	Δρ_min_ = −0.16 e Å^−^^3^
0 restraints	Extinction correction: *SHELXL97* (Sheldrick, 2008), Fc^*^=kFc[1+0.001xFc^2^λ^3^/sin(2θ)]^-1/4^
Primary atom site location: structure-invariant direct methods	Extinction coefficient: 0.024 (3)

### Special details

**Table:**

Geometry. All e.s.d.'s (except the e.s.d. in the dihedral angle between two l.s. planes) are estimated using the full covariance matrix. The cell e.s.d.'s are taken into account individually in the estimation of e.s.d.'s in distances, angles and torsion angles; correlations between e.s.d.'s in cell parameters are only used when they are defined by crystal symmetry. An approximate (isotropic) treatment of cell e.s.d.'s is used for estimating e.s.d.'s involving l.s. planes.
Refinement. Refinement of *F*^2^ against ALL reflections. The weighted *R*-factor *wR* and goodness of fit *S* are based on *F*^2^, conventional *R*-factors *R* are based on *F*, with *F* set to zero for negative *F*^2^. The threshold expression of *F*^2^ > σ(*F*^2^) is used only for calculating *R*-factors(gt) *etc*. and is not relevant to the choice of reflections for refinement. *R*-factors based on *F*^2^ are statistically about twice as large as those based on *F*, and *R*- factors based on ALL data will be even larger.

### Fractional atomic coordinates and isotropic or equivalent isotropic displacement parameters (Å^2^)

**Table:**

	*x*	*y*	*z*	*U*_iso_*/*U*_eq_	
N1	0.6956 (2)	0.7443 (3)	0.35815 (9)	0.0470 (5)	
N2	0.77449 (17)	0.4023 (3)	0.46730 (8)	0.0410 (5)	
H21	0.8406	0.5181	0.4539	0.061*	
C5	0.5911 (2)	0.5707 (4)	0.37611 (10)	0.0389 (5)	
C6	0.6318 (2)	0.3877 (4)	0.43697 (10)	0.0391 (5)	
N4	0.74724 (18)	0.0445 (3)	0.54961 (8)	0.0438 (5)	
H41	0.6579	−0.0130	0.5370	0.066*	
N5	0.83128 (19)	−0.0771 (3)	0.60411 (9)	0.0481 (5)	
N3	0.96153 (18)	0.2595 (3)	0.55752 (9)	0.0454 (5)	
C3	0.4141 (3)	0.7178 (5)	0.28387 (12)	0.0529 (7)	
H3	0.3198	0.7083	0.2589	0.063*	
C4	0.4492 (2)	0.5512 (4)	0.34098 (11)	0.0478 (6)	
H4	0.3789	0.4286	0.3555	0.057*	
C1	0.6574 (3)	0.9042 (4)	0.30331 (12)	0.0547 (6)	
H1	0.7281	1.0284	0.2905	0.066*	
C7	0.8255 (2)	0.2395 (4)	0.52329 (11)	0.0380 (5)	
C2	0.5196 (3)	0.8965 (5)	0.26449 (12)	0.0534 (7)	
H2	0.4990	1.0104	0.2260	0.064*	
C8	0.9568 (2)	0.0599 (4)	0.60561 (11)	0.0490 (6)	
H8	1.0385	0.0229	0.6379	0.059*	
O1	0.53795 (16)	0.2298 (3)	0.45747 (8)	0.0520 (5)	

### Atomic displacement parameters (Å^2^)

**Table:**

	*U*^11^	*U*^22^	*U*^33^	*U*^12^	*U*^13^	*U*^23^
N1	0.0448 (11)	0.0521 (13)	0.0439 (10)	−0.0024 (9)	−0.0056 (8)	0.0052 (10)
N2	0.0348 (10)	0.0466 (12)	0.0414 (10)	−0.0067 (8)	−0.0051 (8)	0.0074 (9)
C5	0.0382 (12)	0.0424 (13)	0.0360 (11)	0.0004 (10)	−0.0015 (9)	−0.0021 (10)
C6	0.0346 (11)	0.0436 (14)	0.0390 (12)	−0.0060 (10)	−0.0031 (9)	−0.0033 (10)
N4	0.0371 (10)	0.0510 (13)	0.0431 (10)	−0.0086 (9)	−0.0042 (8)	0.0068 (9)
N5	0.0419 (10)	0.0541 (13)	0.0481 (10)	−0.0055 (10)	−0.0077 (8)	0.0132 (9)
N3	0.0391 (10)	0.0500 (12)	0.0469 (10)	−0.0077 (9)	−0.0092 (8)	0.0089 (9)
C3	0.0490 (14)	0.0611 (18)	0.0482 (13)	0.0082 (12)	−0.0150 (11)	−0.0033 (12)
C4	0.0438 (13)	0.0529 (15)	0.0465 (13)	−0.0028 (11)	−0.0065 (10)	−0.0015 (11)
C1	0.0580 (15)	0.0517 (16)	0.0542 (14)	−0.0055 (12)	−0.0027 (11)	0.0127 (12)
C7	0.0320 (11)	0.0441 (14)	0.0379 (11)	−0.0058 (10)	−0.0014 (9)	−0.0008 (10)
C2	0.0620 (16)	0.0542 (17)	0.0439 (12)	0.0088 (13)	−0.0074 (11)	0.0059 (12)
C8	0.0438 (13)	0.0582 (16)	0.0447 (13)	−0.0034 (12)	−0.0128 (10)	0.0109 (12)
O1	0.0390 (9)	0.0594 (11)	0.0574 (10)	−0.0141 (8)	−0.0100 (7)	0.0117 (8)

### Geometric parameters (Å, °)

**Table:**

N1—C5	1.333 (2)	N5—C8	1.309 (3)
N1—C1	1.333 (3)	N3—C7	1.329 (2)
N2—C6	1.349 (2)	N3—C8	1.362 (2)
N2—C7	1.388 (2)	C3—C2	1.364 (3)
N2—H21	0.8751	C3—C4	1.379 (3)
C5—C4	1.381 (3)	C3—H3	0.9300
C5—C6	1.495 (3)	C4—H4	0.9300
C6—O1	1.226 (2)	C1—C2	1.377 (3)
N4—C7	1.324 (2)	C1—H1	0.9300
N4—N5	1.371 (2)	C2—H2	0.9300
N4—H41	0.8612	C8—H8	0.9300
			
C5—N1—C1	116.71 (19)	C4—C3—H3	120.4
C6—N2—C7	122.55 (17)	C3—C4—C5	118.5 (2)
C6—N2—H21	122.2	C3—C4—H4	120.7
C7—N2—H21	115.2	C5—C4—H4	120.7
N1—C5—C4	123.29 (19)	N1—C1—C2	124.0 (2)
N1—C5—C6	117.68 (18)	N1—C1—H1	118.0
C4—C5—C6	119.02 (19)	C2—C1—H1	118.0
O1—C6—N2	122.01 (19)	N4—C7—N3	110.85 (18)
O1—C6—C5	120.34 (18)	N4—C7—N2	125.30 (17)
N2—C6—C5	117.65 (18)	N3—C7—N2	123.85 (18)
C7—N4—N5	110.26 (16)	C3—C2—C1	118.4 (2)
C7—N4—H41	130.3	C3—C2—H2	120.8
N5—N4—H41	119.4	C1—C2—H2	120.8
C8—N5—N4	101.06 (17)	N5—C8—N3	116.56 (18)
C7—N3—C8	101.28 (17)	N5—C8—H8	121.7
C2—C3—C4	119.1 (2)	N3—C8—H8	121.7
C2—C3—H3	120.4		
			
C1—N1—C5—C4	0.0 (3)	C5—N1—C1—C2	−1.0 (3)
C1—N1—C5—C6	179.44 (19)	N5—N4—C7—N3	−0.1 (2)
C7—N2—C6—O1	0.4 (3)	N5—N4—C7—N2	179.57 (17)
C7—N2—C6—C5	−178.98 (17)	C8—N3—C7—N4	0.3 (2)
N1—C5—C6—O1	177.46 (18)	C8—N3—C7—N2	−179.40 (19)
C4—C5—C6—O1	−3.1 (3)	C6—N2—C7—N4	4.8 (3)
N1—C5—C6—N2	−3.1 (3)	C6—N2—C7—N3	−175.51 (19)
C4—C5—C6—N2	176.30 (17)	C4—C3—C2—C1	−0.4 (3)
C7—N4—N5—C8	−0.1 (2)	N1—C1—C2—C3	1.2 (4)
C2—C3—C4—C5	−0.5 (3)	N4—N5—C8—N3	0.3 (2)
N1—C5—C4—C3	0.7 (3)	C7—N3—C8—N5	−0.4 (2)
C6—C5—C4—C3	−178.68 (19)		

### Hydrogen-bond geometry (Å, °)

**Table:**

*D*—H···*A*	*D*—H	H···*A*	*D*···*A*	*D*—H···*A*
N2—H21···N3^i^	0.88	2.09	2.946 (2)	164
N4—H41···O1^ii^	0.86	2.06	2.873 (2)	158
N4—H41···O1	0.86	2.17	2.629 (2)	113

Symmetry codes: (i) −*x*+2, −*y*+1, −*z*+1; (ii) −*x*+1, −*y*, −*z*+1.
